# Racial Disparities in Plasma Cytokine and Microbiome Profiles

**DOI:** 10.3390/microorganisms12071453

**Published:** 2024-07-17

**Authors:** Kevin D. Fan, Elizabeth Ogunrinde, Zhuang Wan, Chao Li, Wei Jiang

**Affiliations:** 1 Department of Microbiology & Immunology, Medical University of South Carolina, 173 Ashley Ave. Basic Science Building BS208F, Charleston, SC 29425, USA; kevindfan@gmail.com (K.D.F.); wanzhu@musc.edu (Z.W.); 2 Department of Biology, Duke University, Durham, NC 27708, USA; 3 Morton Plant North Bay Hospital, Lutz, FL 34652, USA; elogunrinde@gmail.com; 4 Oklahoma State University Center for Health Sciences, Tulsa, OK 74106, USA; chao.li@okstate.edu; 5 Ralph H. Johnson VA Medical Center, Charleston, SC 29401, USA

**Keywords:** microbiome, racial disparities, plasma cytokine, IL-6

## Abstract

Background: Many health issues prevalent in African American (AA) populations are associated with chronic inflammation and related health conditions, including autoimmune diseases, infectious diseases, neurologic disorders, metabolic syndromes, and others. The current study aims to understand plasma microbiome translocation as a potential trigger for chronic inflammation. Methods: In this study, 16 Caucasian American (CA) and 22 African American (AA) healthy individuals were recruited. Microbial DNA was isolated from the plasma samples and sequenced via microbial 16S rRNA V3-4 sequencing. The plasma levels of 33 cytokines and chemokines were evaluated. The proinflammatory microbiomes were verified using human THP-1 cells in vitro. Results: The plasma levels of IL-6, IL-15, MIP-1α, MIP-1β, and MIP-3α were higher in the AA people, whereas IL-1α and IL-27 were elevated in the CA people. The plasma microbiomes exhibited eight bacterial genera/phyla differentially enriched in the CA and AA people. Given the critical role of IL-6 in chronic inflammation and associated diseases, we identified five bacteria genera significantly associated with IL-6. The abundance of Actinomyces was positively correlated with the plasma IL-6 level (r = 0.41, *p* = 0.01), while the abundance of *Kurthia* (r = −0.34, *p* = 0.04), *Noviherbaspirillum* (r = −0.34, *p* = 0.04), *Candidatus Protochlamydia* (r = −0.36, *p* = 0.03), and *Reyranella* (r = −0.39, *p* = 0.02) was negatively correlated with this. Finally, the THP-1 cells treated with heat-killed bacteria produced higher levels of IL-6 in vitro in response to the Actinomyces species compared to the species in the genus either uncorrelated or negatively correlated with IL-6. Conclusions: This is the first study to report potential blood microbiome translocation as a driver for persistently elevated IL-6 levels in the periphery in healthy AA versus CA people. Understanding the plasma microbiome linked to the IL-6 levels in people with different racial backgrounds is essential to unraveling the therapeutic approaches to improve precision medicine.

## 1. Introduction

African American (AA) populations exhibit a higher incidence of chronic inflammation and related health conditions, including autoimmune diseases, infectious diseases, neurologic disorders, cancer development, metabolic syndromes, and others. It has been demonstrated that AA people often have elevated levels of inflammatory mediators, particularly interleukin (IL)-6 [1,2,3,4]. IL-6 plays important roles in immune homeostasis and the pathogenesis of autoimmune diseases, such as rheumatoid arthritis (RA) and systemic lupus erythematosus (SLE), as well as in infectious diseases like sepsis and SARS-CoV2 pneumonia [5]. Additionally, IL-6 is associated with stroke risk and cancer development [1,3,4]. Therefore, it is critical to understand the cause of elevated IL-6 levels and/or other inflammatory mediators in the AA group. Racial differences have been observed in autoimmune diseases, sepsis, stroke, multiple sclerosis (MS), cancers, and metabolic syndromes. The recent studies have revealed doubled rates of SLE among AA patients [6], with lupus nephritis disproportionately affecting minorities, particularly AA individuals, leading to higher rates of the progression to end-stage kidney disease [7]. Furthermore, AA people experiencing septic shock exhibit higher rates of end-stage renal disease and hypertension compared to their Caucasian American (CA) counterparts. Although the mortality rate did not significantly differ between the groups, the AA patients with septic shock necessitated higher doses and a longer duration of treatment compared to those of the CA patients [8]. AA people also face elevated rates of stroke [1,4], MS [9], and more aggressive breast cancer diagnoses [3]. Moreover, hypertension among AA people is more common, severe, and resistant to treatment compared to that among CA people [10]. Although the racial differences in this inflammation-related disease have been widely observed, the underlying mechanisms are poorly understood. It is important to investigate the risk factors that contribute to this chronic inflammation, which is believed to contribute to the development of many inflammation-related diseases.

Various factors contribute to the higher prevalence of autoimmune diseases among AA people, including exposure to lifetime stressors, systemic inflammation, and altered microbiome compositions. Rheumatoid arthritis, for instance, has been associated with changes in the oral and gut microbiomes, which can exacerbate inflammatory events, contributing to joint damage [11]. Ethnicity has been identified as a crucial determinant of oral microbial colonization, with AA people exhibiting significantly less bacterial diversity and equitability compared to that of people of other ethnicities [12]. AA people also show distinct oral microbial compositions characterized by a higher abundance of certain pathogens [13]. Similarly, disparities are evident in the gut [14], vaginal [15], skin [16], and oral [13] microbiomes among different racial groups. Regarding neurological disorders, studies have linked microbiota disruption with various impairments, including cognitive function, memory formation, and changes in behavior. Short-chain fatty acids derived from the microbiota influence the function of brain immune cells [17]. These data suggest that the microbiome differences may be linked to different lifestyles, such as diet and dietary supplement use, exercise levels, smoking status, social stress levels, and chronic inflammation. Each individual has a varied lifestyle, which is hard to control. However, all of these different lifestyles can seemingly be reflected in microbiome alterations. Altered microbiome levels may directly contribute to the chronic inflammatory response. Therefore, understanding the microbiome disparities among different racial groups may expand our knowledge of the cause-and-result relationship among lifestyle/microbiome/chronic inflammation.

Despite the previous investigations into racial disparities in microbiomes, the plasma microbiome profiles between AA and CA people remain to be explored. Our previous studies suggest that the plasma microbiome profiles are distinct from the gut microbiomes [18]. The plasma microbiome directly interacts with immune cells, which may play a critical role in the chronic inflammatory response. The dynamic alteration of the plasma microbiome may reflect lifestyle changes and contribute to the health of individuals. Additionally, the association between plasma cytokine/chemokine profiles and microbiome profiles in these populations requires further investigation. This study aims to analyze the plasma cytokine and chemokine profiles, along with the microbiome profiles, in healthy AA and CA people to elucidate the bacterial populations contributing to differences in the cytokine/chemokine profiles between the two groups.

## 2. Materials and Methods

### 2.1. Human Subjects

This study was approved by the Institutional Review Board (IRB) of the Medical University of South Carolina. We recruited healthy pre- and post-menopausal females through an advertisement. All the recruited participants for this study provided written consent. In the present study, 22 healthy African American (AA) and 16 healthy Caucasian American (CA) people were included. The inclusion criteria were: (1) men and women aged 18 or older, (2) capable and willing to provide informed consent, and (3) willing to provide saliva and blood samples. The exclusion criteria were as follows: (1) being pregnant or breastfeeding according to a self-report; (2) recently having had a severe illness, such as anemia, acute infections, or cancer; (3) requiring or using specific medications within 120 days before enrollment (antibiotics, systemic immunomodulatory agents, or supraphysiologic doses of steroids (>10 mg/day)); and (4) taking any other condition determined by the investigator to render the subject unsuitable for participation in the study or unable to comply with study requirements. The demographic characteristics of cohort are listed in Table 1.

### 2.2. Plasma Sample Collection

Plasma samples were isolated from the non-fasting volunteers using EDTA-containing blood collection tubes at the Medical University of South Carolina, aliquoted, and stored at −80 °C before use following previously described methods [18].

### 2.3. Plasma Circulating Microbial 16S rDNA V3-4 Sequencing

Plasma 16S rRNA analysis was conducted following a previously described protocol [19]. In the current study, bacterial DNA was isolated from 400 μL of plasma using the QIAamp UCP Pathogen Mini Kit (Qiagen, Hilden, Germany). Endotoxin-free water was used as a control. To maintain consistency, all the samples were sequenced simultaneously. The V4 variable region of the bacterial 16S rDNA gene was amplified using PCR primers 515/806 in HotStarTaq Plus Master Mix (Qiagen) under the following conditions: 94 °C for 3 min, followed by 28 cycles of 94 °C for 30 s, 53 °C for 40 s, and 72 °C for 1 min, with a final elongation step at 72 °C for 5 min. Sequencing was performed on an Ion Torrent PGM at MR DNA (Shallowater, TX, USA). A proprietary analysis pipeline (MR DNA) was used to process the sequencing data. Briefly, the sequences were depleted of barcodes and primers, sequences less than 200 bp were removed, and sequences with ambiguous base calls and homopolymer runs exceeding 6 bp were also excluded. The sequences were denoised, and operational taxonomic units (OTUs) were defined by clustering at 97% similarity, followed by the removal of singleton sequences and chimeras. The final OTUs were taxonomically classified using BLASTn against a database derived from RDPII and NCBI. OTU tables and different taxonomic tables were imported into R for statistical analysis. To eliminate potential bacterial 16S rDNA contamination from the reagents, we applied the strategies for eliminating background and potential artifacts from plasma microbiomes and removed genera detected in the water controls from the samples at the OTU level as described in our previous study [20].

### 2.4. Plasma Levels of Inflammatory Biomarkers

All plasma assays were conducted in accordance with the manufacturer’s protocol for the Human Chemokine Panel 1 “V-PLEX Plus” and Human Proinflammatory Panel kits (Meso Scale Diagnostics, Rockville, MD, USA). Subsequently, each multiplex array underwent scanning using an MESO QuickPlex SQ 120. The analysis of arrays and the quantification of biomarker concentrations were performed utilizing Discover Workbench 4.0 software (Meso Scale Diagnostics), which employed the manufacturer’s supplied standards to generate standard curves.

### 2.5. Bacterial Culture

*Phyllobacterium myrsinacearum*, *Kurthia gibsonii*, *Noviherbaspirillum autotrophicum*, and *Actinomyces naeslundii* were purchased from ATCC (Manassas, VA, USA). Briefly, *Phyllobacterium myrsinacearum* (strain: NCIB 12127, ATCC) was grown in nutrient broth at 26 °C in a shaker incubator. *Kurthia gibsonii* (strain: NCIB 9758 [S8], ATCC) was cultured aerobically in brain heart infusion broth (Thermo Fisher Scientific, Waltham, MA, USA) at 30 °C in a shaker incubator. *Noviherbaspirillum autotrophicum* (strain: TSA66, ATCC) was cultured aerobically in R2A medium (NEOGEN, Lexington, KY, USA) at 30 °C in a shaker incubator. *Actinomyces naeslundii* (strain: 279 [CDC W826, NCTC 10301], ATCC) was cultured under anaerobic conditions in modified chopped meat medium (Hardy Diagnostics, Santa Maria, CA, USA) at 37 °C. For subsequent experiments, each bacterium was heat-inactivated at 60 °C for 30 min, followed by centrifugation at 6000 rpm for 10 min. The resulting pellet was suspended in PBS, and the bacterial count was quantified using a Quantom TX microbial cell counter (Logos Biosystems, Annandale, VA, USA).

### 2.6. Cell Culture and Incubation with Bacteria

Human monocyte cell line THP-1 cells were obtained from ATCC (Manassas, VA, USA). The cells were cultured at a density of 2–6 × 10^5^ cells/mL in RPMI 1640 medium supplemented with heat-inactivated 10% fetal bovine serum (FBS, Gibco, Thermo Fisher Scientific, Waltham, MA, USA) and 1% penicillin/streptomycin (Gibco, Thermo Fisher Scientific, Waltham, MA, USA), and maintained at 37 °C in a 5% CO_2_ incubator. The experiments were conducted using THP-1 cells within the 6th passage.

For the subsequent experiments, THP-1 cells in RPMI 1640 medium containing 10% FBS, but without antibiotics, were seeded into a 12-well plate at a density of 6 × 10^6^ cells/mL/well. Heat-inactivated *Phyllobacterium myrsinacearum*, *Kurthia gibsonii*, *Noviherbaspirillum autotrophicum*, and *Actinomyces naeslundii* were added into the cell culture at a final concentration of 1 × 10^7^ CFU/mL/well and incubated for 12 and 24 h. The supernatants were collected for ELISA analysis.

### 2.7. Enzyme-Linked Immunosorbent Assay (ELISA)

Human IL-6 levels in the supernatant were measured with a human IL-6 ELISA kit (Invitrogen, Thermo Fisher Scientific, Waltham, MA, USA) according to the manufacturer’s protocol.

### 2.8. Statistical Analysis

Non-parametric Mann–Whitney’s U tests were performed to compare the differences in continuous measurements between groups using GraphPad Prism software version 9. Spearman’s rank correlation was used to explore the associations between pairs of continuous variables. The data are expressed as median with 95% CI. For the ELISA results, statistical significance was determined by analysis of variance (ANOVA) with Fisher’s probable least-squares difference test using GraphPad Prism software. The data are expressed as means ± standard error of the mean. The alpha diversity of richness and evenness was calculated using the Simpson diversity index for each sample. The Unifrac coefficient was calculated to evaluate beta diversity and compositional dissimilarity among the microbial community. A value of *p* < 0.05 was considered statistically significant.

## 3. Results

### 3.1. Plasma Cytokine/Chemokine Levels Were Differentially Expressed in the CA and AA Groups

**Figure 1:**
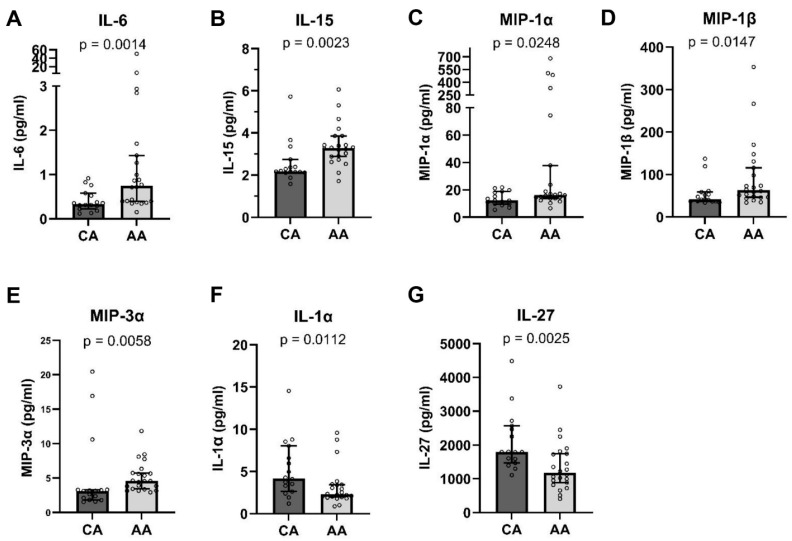
Plasma cytokine/chemokine levels were differential expressed in AA and CA people. Plasma cytokine and chemokine levels from AA (N = 22) and CA (N = 16) people were analyzed. Seven differentially expressed cytokines and chemokines, including IL-6 (**A**), IL-15 (**B**), MIP-1α (**C**), MIP-1β (**D**), MIP-3α (**E**), IL-1α (**F**), and IL-27 (**G**), are shown. Data are expressed as median with 95% CI, and *p* < 0.05 was considered statistically significant.

The plasma cytokine and chemokine levels were determined and analyzed between the CA and AA groups. Our data reveal significant differences in the expression of seven cytokines between the CA and AA people. IL-6 (Figure 1A, *p* < 0.01), IL-15 (Figure 1B, *p* < 0.01), MIP-1α (Figure 1C, *p* < 0.05), MIP-1β (Figure 1D, *p* < 0.05), and MIP-3α (Figure 1E, *p* < 0.01) exhibited higher levels in the AA people, whereas IL-1α (Figure 1F, *p* < 0.05) and IL-27 (Figure 1G, *p* < 0.01) were elevated in the CA people. These data suggest that the AA group exhibited higher levels of plasma inflammatory mediators. Especially, elevated plasma IL-6 levels may be associated with autoimmune and infectious diseases.

### 3.2. Plasma Bacteria Profiles Were Altered Considerably between the CA and AA Groups

**Figure 2:**
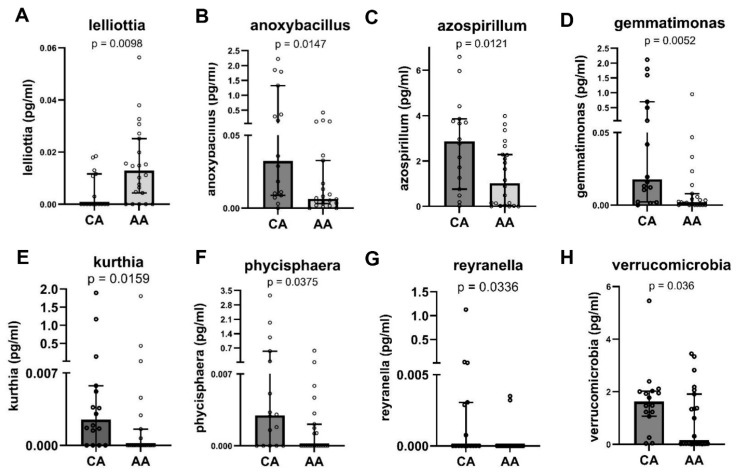
Plasma bacterial profiles were differential expressed in AA and CA people. Plasma bacteria levels from AA (N = 22) and CA (N = 16) people were analyzed. Differing levels of eight bacteria, including *Lelliotta* spp. (**A**), *Anoxybacillus* spp. (**B**), *Azospirillum* spp. (**C**), *Gemmatimonas* spp. (**D**), *Kurthia* spp. (**E**), *Phycisphaera* spp. (**F**), *Reyranella* spp. (**G**), and *Verrucomicrobia* (**H**), between AA and CA people are shown. Data are expressed as median with 95% CI, and *p* < 0.05 was considered statistically significant.

The plasma bacteria levels were determined and analyzed between the CA and AA groups. We demonstrated that eight bacterial genera/species were differentially expressed in the CA and AA people; *Lelliotta* spp. (Figure 2A, *p* < 0.01) exhibited higher levels in the AA people, whereas *Anoxybacillus* spp. (Figure 2B, *p* < 0.05), *Azospirillum* spp. (Figure 2C, *p* < 0.05), *Gemmatimonas* spp. (Figure 2D, *p* < 0.01), *Kurthia* spp. (Figure 2E, *p* < 0.05), *Phycisphaera* spp. (Figure 2F, *p* < 0.05), *Reyranella* spp. (Figure 2G, *p* < 0.05), and *Verrucomicrobia* (Figure 2H, *p* < 0.05) were elevated in the CA people. These data demonstrated that similar to the gut, vaginal, skin, and oral microbiomes, the plasma microbiomes exhibit racial differences between AA and CA groups.

### 3.3. The Association between Plasma Cytokine/Chemokine Levels and Bacteria Profiles

**Figure 3:**
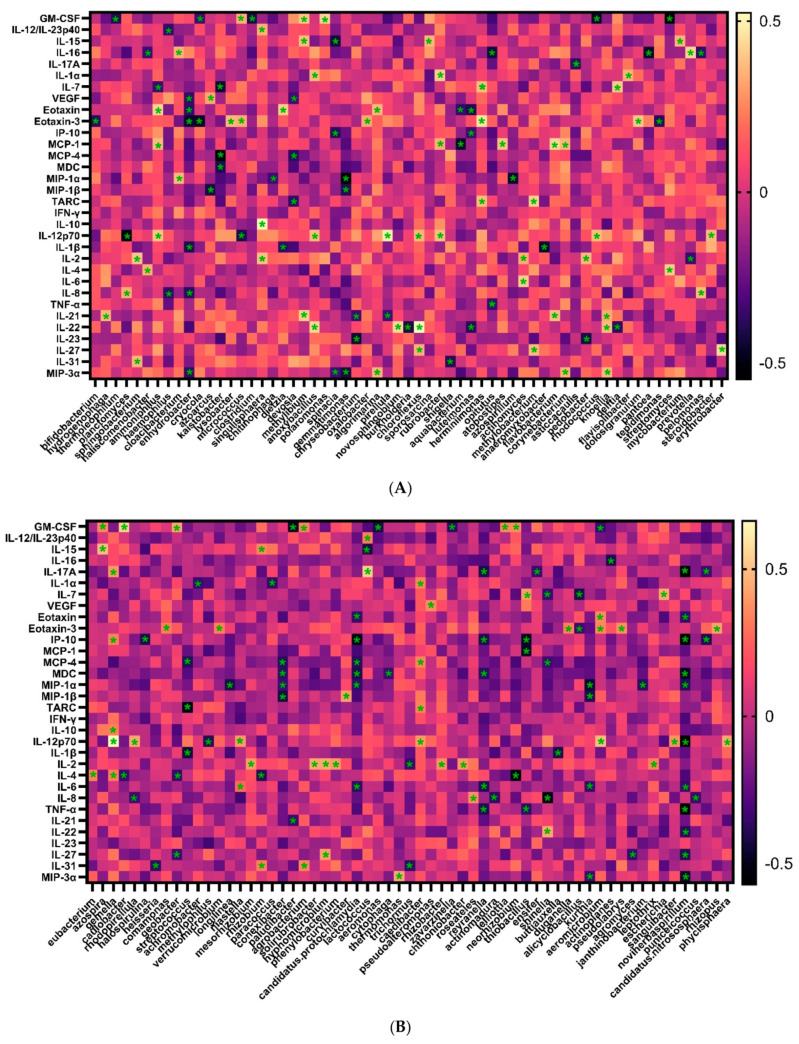

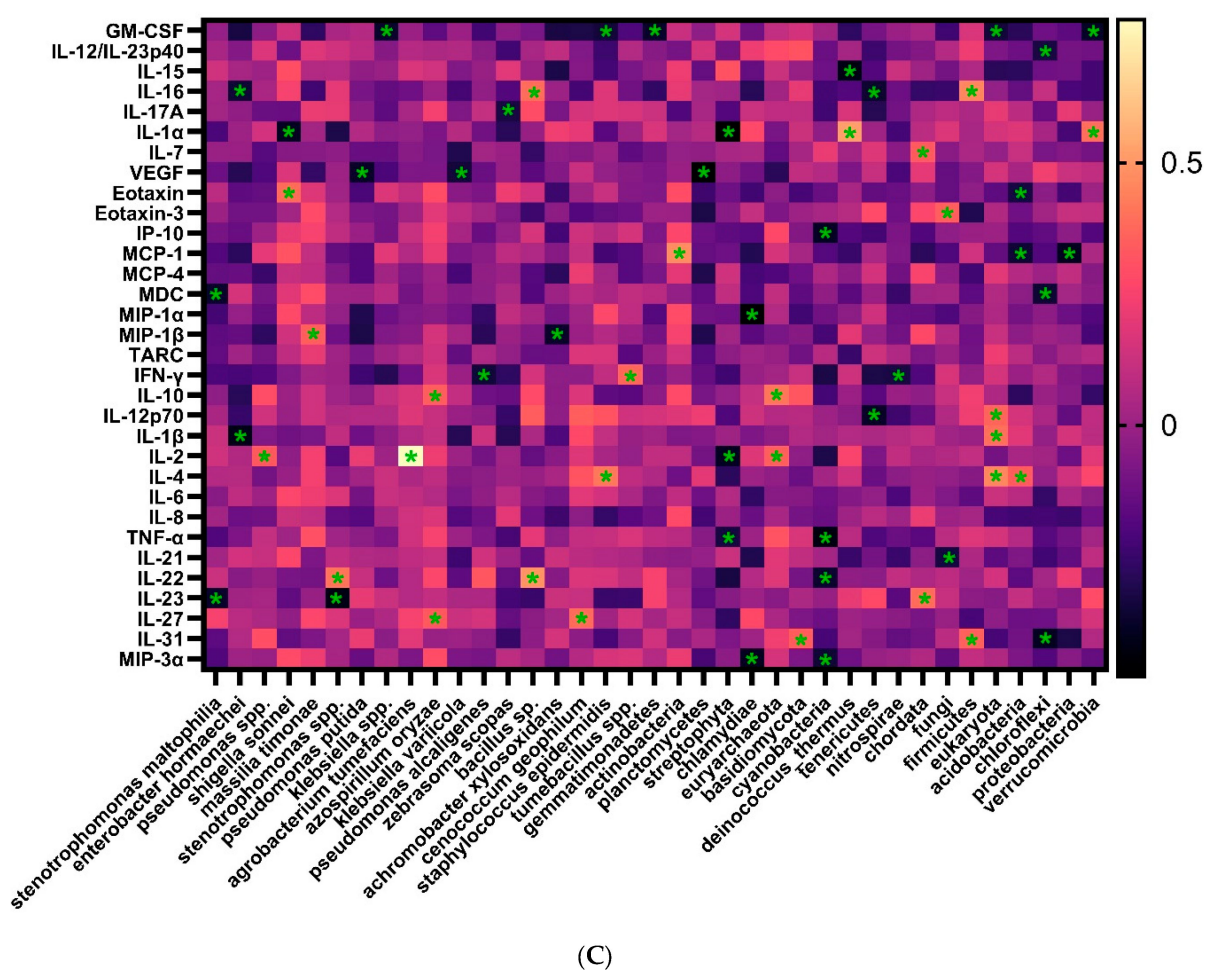
The association between plasma cytokine/chemokine levels and bacterial profiles. The association between plasma cytokine/chemokine levels and bacterial profiles from AA (N = 22) and CA people (N = 16) were analyzed. One hundred and twenty-two bacterial genera (**A**,**B**) and thirty-seven bacterial species (**C**) were associated with cytokines/chemokines. The asterisk indicates significant association, the lighter color indicates positive association, and the darker color indicates negative association. *p* < 0.05 was considered statistically significant.

We next determined the association between the plasma cytokine/chemokine levels using Spearman’s correlation test. We observed that the cytokine/chemokine levels were differentially associated with 122 bacterial genera (Figure 3A,B, *p* < 0.05) and 37 bacterial species (Figure 3C, *p* < 0.05). These data suggested that the plasma bacteria profiles are associated with the cytokine and chemokine levels.

### 3.4. Bacteria Associated with Plasma IL-6 Levels and Their Effects on IL-6 Production in THP-1 Cells

**Figure 4:**
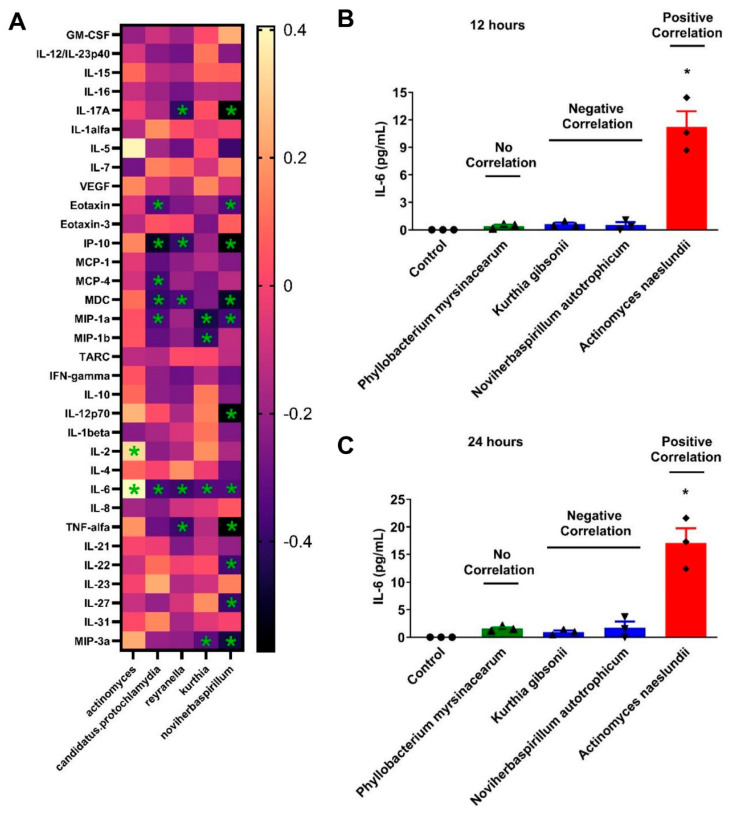
The bacteria associated with the plasma IL-6 levels and their effects on IL-6 produced by THP-1 cells. The association between the plasma IL-6 levels and bacteria profiles from AA (N = 22) and CA people (N = 16) was analyzed (**A**). The asterisk indicates a significant association, the lighter color indicates a positive association, and the darker color indicates a negative association. Heat-killed *Phyllobacterium myrsinacearum* (bacteria with no correlation with IL-6), *Kurthia gibsonii*, *Noviherbaspirillum autotrophicum* (bacteria negatively correlated with IL-6), and *Actinomyces naeslundii* (bacteria positively correlated with IL-6) were incubated with the THP-1 cells for 12 (**B**) and 24 (**C**) h, and the IL-6 levels were determined. The data are expressed as means ± standard error of the mean. N = 3 independent experiments. *p* < 0.05 was considered statistically significant.

Since the plasma IL-6 levels have been shown to be associated with autoimmune diseases and infectious diseases [5], we specifically analyzed the bacteria associated with the IL-6 levels. We identified five bacterial genera significantly associated with IL-6; Actinomyces are positively correlated with IL-6 (r = 0.41, *p* = 0.01), while *Kurthia* (r = −0.34, *p* = 0.04), *Noviherbaspirillum* (r = −0.34, *p* = 0.04), *Candidatus Protochlamydia* (r = −0.36, *p* = 0.03), and *Reyranella* (r = −0.39, *p* = 0.02) are correlated negatively (Figure 4A, *p* < 0.05). To confirm that the bacterial abundance with a positive or negative correlation with IL-6 have differential effects on human monocyte activation, we cultured THP-1 cells and *Phyllobacterium myrsinacearum* (bacteria with no correlation with IL-6), *Kurthia gibsonii*, *Noviherbaspirillum autotrophicum* (bacteria negatively correlated with IL-6), and *Actinomyces naeslundii* (bacteria positively correlated with IL-6) for 12 and 24 h. Our data demonstrated that heat-killed *A. naeslundii* treated monocytes produced significantly higher IL-6 compared to the either uncorrelated or negatively correlated species (Figure 4B,C, *p* < 0.05, N = 3). These results confirm that the plasma specific bacterial DNA translocation can contribute to cytokine/chemokine production in the circulation.

## 4. Discussion

In the current study, we analyzed the plasma microbiomes and correlations with blood inflammation in AA and CA people and found that there were several microbiome differences in the AA and CA people that were positively or negatively correlated to the plasma IL-6 levels. The microbiome can modulate host immunity [21]. Many studies have analyzed the microbiomes in stools or in samples from other mucosal sites that may not represent the systemic microbiome, and thus may not play a direct role in the systemic environment and systemic chronic inflammatory diseases. Here, we utilized cutting-edge research methods published by our colleagues and us, including blood and tissue microbiome analysis and the direct measurement of microbial product translocation [19,20,22,23,24,25]. Analyzing the levels of bacterial 16S rRNA and the microbiome in plasma is challenging due to contamination and stringent technical demands. We have performed this for more than ten years and have published several papers on this subject [18].

Our findings affirmed previous research, indicating higher levels of IL-6 in the plasma of the AA individuals compared to those of the CA people [26,27]. In addition, we identified elevated levels of plasma IL-15, MIP-1α, MIP-1β, and MIP-3α in the AA people compared to the CA people. IL-15 plays a critical role in the development and maintenance of various immune cells such as T cells, suggesting elevated T cell activation in AA people, potentially contributing to autoimmune diseases [28]. Elevated MIP levels also indicate increased monocyte activation in AA individuals. Interestingly, we observed decreased levels of IL-1α and IL-27 in the AA people compared to those of the CA people. Since both IL-1α and IL-27 regulate T cell activation [29,30], altered IL-1α and IL-27 levels may suggest altered T cell immunity between the two racial groups.

Recent studies have shown that commensal bacteria in the gut translocate to the system and drive autoimmunity [31,32,33]. While the racial differences in the gut [14], vaginal [15], skin [16], and oral [13] microbiomes have been previously studied, the differences in plasma microbiomes remain unexplored. Our analysis revealed the differential expression of eight bacterial genera between the CA and AA people. *Lelliotta* spp. exhibited higher levels in the AA people, whereas the levels of *Anoxybacillus* spp., *Azospirillum* spp., *Gemmatimonas* spp., *Kurthia* spp., *Phycisphaera* spp., *Reyranella* spp., and *Verrucomicrobia* were elevated in the CA people. To elucidate the relationship between altered cytokine/chemokine levels and bacterial levels, we investigated the association between the plasma microbiome and cytokine levels. Our data suggest a direct interaction between the plasma microbiome and immune cells, dynamically regulating the inflammatory response. Confirming this hypothesis, we cultured human monocyte cell line THP-1 cells with bacteria positively, negatively, or not correlated with IL-6. The bacteria associated with IL-6 induced higher IL-6 production in the THP-1 cells, underscoring the importance of analyzing the plasma microbiome profiles. Understanding the microbiome profiles and potentially influencing the plasma microbiome through interventions such as dietary changes may significantly alter the inflammatory cytokine and chemokine levels, thereby regulating T cell activation and disease development. To further explore the differences in plasma microbiome between AA and CA people, we analyzed the alpha and beta diversities, finding no significant differences (Supplementary Figures S1 and S2).

A recent study revealed racial differences in the plasma microbiomes in patients with systemic lupus erythematosus (SLE) [34]. In this study, the AA patients demonstrated differences in plasma bacterial presence, including more genus microbiomes (i.e., inflammatory bacteria *Burkholderia*) and fewer genus microbiomes (i.e., *Azospirillum*) compared to those of the CA patients. The AA patients had higher SLE Disease Activity Index (SLEDAI) scores and urinary protein levels as well as a trend for increased anti–double-stranded DNA (dsDNA) antibody levels.

We acknowledge the limitations of our study, including the relatively small sample size, which may limit the robustness of our findings. Additionally, low levels of certain bacteria in the plasma pose challenges for analysis, and limitations in the accuracy of the microbial 16s RNA sequencing of the species and strain levels and culture methods restrict our ability to draw a further conclusion. We also want to point out several strengths of this study, including our long-term interest and expertise in the plasma microbiome profile. Analyzing plasma microbiomes is challenging due to contamination and stringent technical demands. However, using bacterial 16S rRNA sequencing, we have successfully developed a method to extract bacterial DNA and subject it to 16S rRNA sequencing. Our track record of publications demonstrates our expertise in this area. In addition, our group has extensive experience in autoimmune diseases such as lupus. We have a deep understanding of the unmet need to characterize the racial differences in inflammatory diseases and their underlying mechanism. In this study, we have not only analyzed the association between the plasma microbiome and inflammatory mediators, but we also cultured individual bacteria and incubated the bacteria with human monocyte cell line THP-1 cells to confirm that the bacteria positively correlated with induced IL-6 production in monocytes.

In summary, this study represents the first investigation into the racial differences in plasma microbiome profiles and the association between plasma cytokine/chemokine levels and microbiome profiles. Understanding plasma cytokine and microbiome levels in diverse racial backgrounds is crucial for advancing precision medicine approaches.

## Figures and Tables

**Table 1:** Demographic characteristics of the cohort.

N = 38	Caucasian American (CA)	African American (AA)
Subject size	16	22
Sex, Females (%)	16 (100%)	22 (100%)
Age ± SEM (years)	41.1 ± 3.7	45.5 ± 3.4
Race (%)		
White	16 (100%)	0 (0%)
Black	0 (0%)	22 (100%)
Other	N/A	N/A

SEM: Standard error of the mean, N/A: Not applicable.

## Data Availability

Data will be made available on reasonable request.

## Supplementary Materials

The following supporting information can be downloaded at: https://www.mdpi.com/article/10.3390/microorganisms12071453/s1, Figure S1: Alpha diversity of plasma microbiome in AA and CA groups. Alpha diversity of plasma microbiome at genus (A) and species (B) levels was compared between AA (N = 22) and CA (N = 16) groups. *p* < 0.05 was considered statistically significant; Figure S2: Beta diversity of plasma microbiome in AA and CA groups. Beta diversity of plasma microbiome at genus (A) and species (B) levels was compared between AA (Black, N = 22) and CA (White, N = 16) groups. *p* < 0.05 was considered statistically significant.
