# Supplementary material for: Racial Disparities in Plasma Cytokine and Microbiome Profiles
Source: Microorganisms. 2024 Jul 17;12(7):1453. doi: 10.3390/microorganisms12071453 (PMC11279229; doi:10.3390/microorganisms12071453)
Supplement: Supplementary file 1 [file microorganisms-12-01453-s001.zip › microorganisms-3016889-supplementary.pdf]

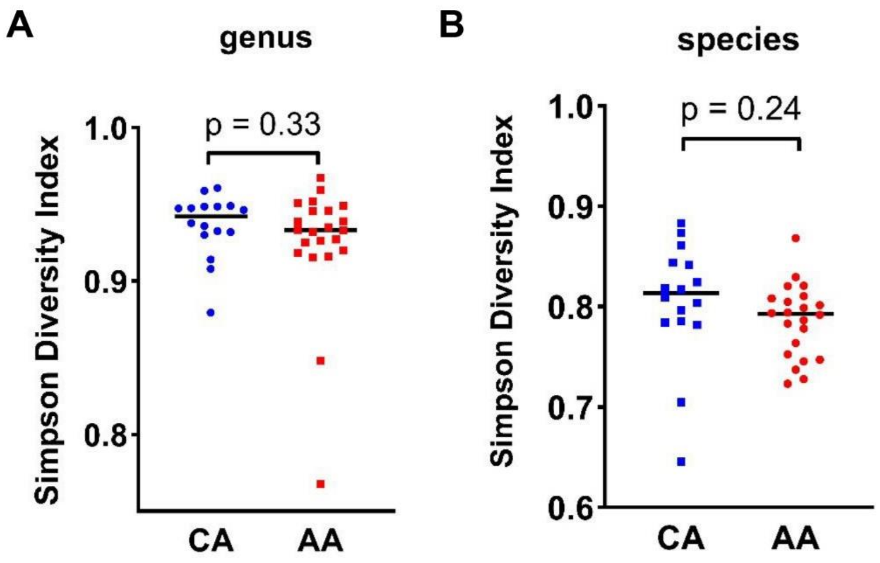

Supplemental Figure S1. Alpha diversity of plasma microbiome in AA and CA groups.

Alpha diversity of plasma microbiome at genus (A) and species (B) levels was compared between AA (N=22) and CA (N=16) groups. $P<0.05$ were considered statistically significant.

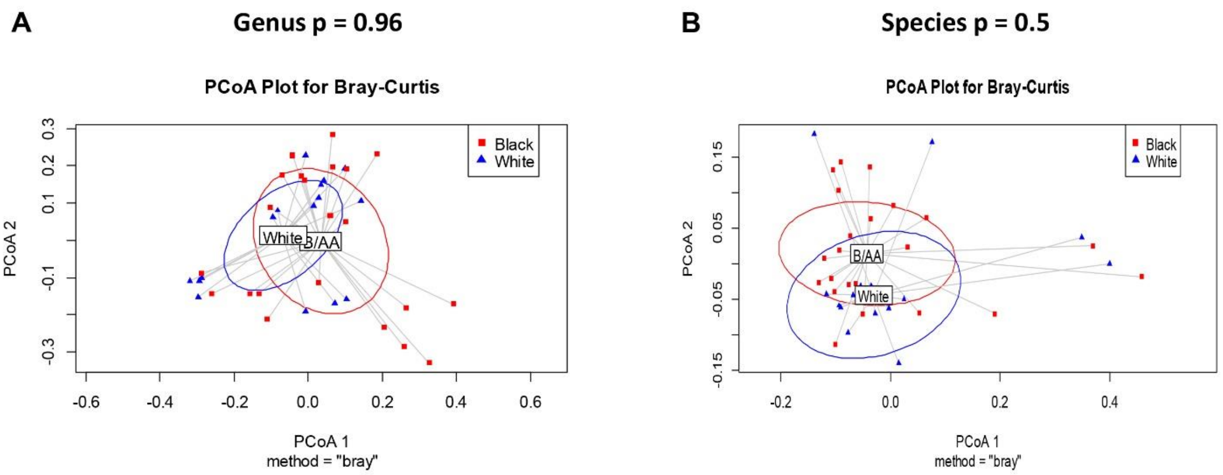

Supplemental Figure S2. Beta diversity of plasma microbiome in AA and CA groups.

Beta diversity of plasma microbiome at genus (A) and species (B) levels was compared between AA (Black, N=22) and CA (White, N=16) groups. P<0.05 were considered statistically significant.
